# Rearing the scuttle fly *Megaselia scalaris* (Diptera: Phoridae) on industrial compounds: implications on size and lifespan

**DOI:** 10.7717/peerj.1085

**Published:** 2015-07-09

**Authors:** Anna Alcaine-Colet, Karl R. Wotton, Eva Jimenez-Guri

**Affiliations:** EMBL/CRG Systems Biology Research Unit, Center for Genomic Regulation (CRG), Barcelona, Spain; Universitat Pompeu Fabra (UPF), Barcelona, Spain

**Keywords:** *Megaselia scalaris*, Modeling clay, Plasticity, Lifespan, Size

## Abstract

*Megaselia scalaris* (Loew, 1866) (Diptera, phoridae) is a cosmopolitan fly species used in forensic science, and has been developed as a laboratory model species. They feed on decaying corpses as well as a wide variety of organic matter, and previous studies have even found them feeding on liquid paint or shoe polish, suggesting the possibility that they could breakdown industrial compounds. To test this possibility, we fed *M. scalaris* on a variety of industrially obtained materials and found that it was unable to complete its life cycle, dying at the larval stage, with the majority of compounds tested. However, when fed on modeling clay, a substrate that contains starch and inedible compounds, it was able to complete its life cycle. On this diet we observed increased larval development time, decreased pupal development time and a shortened adult life span. Additionally, pupae and adult flies were smaller than control flies. Contrary to previous reports, we find no evidence that *M. scalaris* is able to survive on modern formulations of liquid paint.

## Introduction

Human made synthetic materials are polluting the environment and it is important to develop efficient methods for their removal (reviewed in Gregory, 2009; Thompson et al., 2009; Teuten et al., 2009). Saprophages are organisms that feed on decaying organic matter and can be used to treat organic waste (Sheppard et al., 1994). The possibility that they could also dispose of synthetic materials could lead to economic alternatives for the cleanup of pollution and the disposal or conversion of unwanted material. One candidate for such a role is the scuttle fly *Megaselia scalaris* (Loew, 1866). *Megaselia scalaris* is in the family Phoridae and has been used in forensic medicine to estimate post mortem intervals (see Disney, 2008 and Varney & Noor, 2010 for reviews of *M. scalaris* biology). It has been studied as a cause of myiasis in humans (Hira et al., 2004; Mazayad & Rifaat, 2005) and for genetics and developmental biology (Sievert, Kuhn & Traut, 1997; Traut, 1994; Willhoeft & Traut, 1990; Willhoeft & Traut, 1995; Wotton, 2014). It can feed on a wide variety of food, ranging from dead or alive animals, fruits, fungi and laboratory foods (Disney, 2008). Importantly, this organism has also been reported to feed on otherwise inedible materials such as shoe polish or paint (Lever, 1944; McCrae, 1967).

The ability of insects to obtain nutrients from different food sources affects their size; well-fed animals are bigger than poorly fed ones (Chapman, 1998). In flies, the size of the larvae at the moment of pupation will determine the size of both the pupae and of the adult. Two terms define the size of the larvae before metamorphosis: the critical weight is the size at which the larvae will start producing the hormones needed to begin metamorphosis (Nijhout & Williams, 1974), while the minimal viable size is the size at which the larvae will be able to survive metamorphosis (Nijhout, 1975). After attaining the critical weight, larvae can still grow if they continue feeding, but failure to feed will not prevent pupation. If larvae are starved before the critical weight, pupation is compromised (Mirth, Truman & Riddiford, 2005).

The objective of this study is to test the ability of *M. scalaris* to complete its life cycle on a variety of synthetic or semi-synthetic products, and to record the influence of the diets on the flies. For all materials used, only modeling clay consistently sustained the life cycle of *M. scalaris*. We studied the effects of a modeling clay diet on *M. scalaris* and found that these flies were smaller and had shorter adult lives than control flies. Their larval and pupal stages also differed from those of the controls. We discuss the possible reasons for these differences in terms of reduced nutritional value of the food and the critical weight and minimal viable size of the flies.

## Materials and Methods

We tested the ability of *M. scalaris* to complete its life cycle on 13 synthetic products (Table 1). For each condition 4 females and 2 males *M. scalaris* ‘Wien’ strain (Mainx, 1964; Johnson, Mertl & Traut, 1988) were transferred into a tube containing the test food source and allowed to mate. A total of 20 replicates for each condition were made. For all experimental conditions, water was added *ad libitum* to prevent dehydration. Controls were fed on standard *Drosophila melanogaster* (Meigen, 1830) fly food (Table 1). Tubes were kept at stable room temperature (22.8 ± 0.8 °C). The materials used to feed the larvae shown in Table 1 have been classified as mouldable (materials that can be shaped and are therefore more deformable, and easier for the larvae to chew on) and non mouldable. We used a modern formulation of the latex based paint described in McCrae (1967), however this is not an exact equivalent of the 1967 formulation. After egg hatching, adults were removed from the tubes. The presence and survival of larvae, pupae or new adults was monitored daily until no more alive larvae or emergence of new flies were observed. We recorded the duration of each stage (larval, pupal or adult) along with pupal and adult sizes (pupal and adult length, pupal width). In some instances, pupae could not be measured due to the location of the pupation site, since moving the pupae would have caused damage. Only data from the pupae that emerged as adult flies was used to make the comparisons, so that their genders could be assessed. Pictures were taken using a Leica MS5 dissecting scope and a Leica EC3 camera and dimensions measured using the ruler function in Photoshop. Statistical analysis to evaluate differences between conditions was performed using the Student’s *T*-test, with a significance level for the associated probability of 0.05. Data on the size, stage duration and statistical values for animals that completed their life cycle can be found in File S1.

**Table 1:** Test materials used to feed *M. scalaris*. The characteristics of the materials in terms of mouldability, as well as their commercial name, when applicable, or composition, when available, are provided.

Material	Characteristics	Commercial name	Composition
Plastic carrier bag	Non-mouldable	NA	NA
Adhesive tape	Non-moludable	Scotch^®^ 810 Magic™ tape (3M, USA)	Synthetic adhesive, matte acetate
Nitrile gloves	Non-moludable	Versatouch Nitrile Gloves (Num. 92-205 Ansell Healthcare, USA)	Nitrile rubber (synthetic rubber copolymer of acrylonitrile and butadiene)
Latex gloves	Non-moludable	Naturflex Latex gloves (Ref. 121353 Barna Import Medica, S.A., Spain)	Latex
Potato starch carrier bag	Non-moludable	NA	NA
Synthetic paint	Mouldable	Titanlux Gloss Synthetic Paint (Color 539 (luminous blue), Industrias Titan, S.A., Spain)	Nafta, hydrocarbons, xilene, butanone-oxima, cobalt bis (2-ethilhexanoate), 2-butoxietanol, 1-metoxi-2-propanol, 1-metil-2-metocietil acetate, (metil-2-metoxietoxi) propanol, etilbenzene.
Latex based paint	Mouldable	La Pajarita Silk latex based paint (L-12 (intense blue) Carlos Grollo S.A., Spain)	
Petroleum jelly	Mouldable	Vaseline^®^ (Unilever, UK)	Hydrocarbons (carbon numbers >25)
White tack	Mouldable	APLI White Tack (Ref. 11803 Apli Paper S.A.U., Spain)	Mix of polymers, oils and carbonates
Chewing gum	Mouldable	Sugar free Orbit spearmint chewing gum (Wrigley Co. S.L.U, Spain)	Gum base, soy lecithin, high intensity non caloric sweeteners, glycerin, mint essential oil, antioxidant BHA
Wax ear plugs	Mouldable	Ototap (removing the outer cotton coating) (CN304261.1 Prophyl center, S.A., Spain)	Vegetable wax, petroleum jelly, pigment
Periphery wax	Mouldable	Surgident Periphery Wax (No 50092189 Heraeus Kulzer, Inc., USA)	Paraffin wax
Modeling clay	Mouldable	Jovi modelling clay Plastilina (blue) (Art. 70 Jovi, S.A., Spain)	White oil, paraffin waxes, stearic acid, metallic stearates, starch (50%), BHT, pigment blue 15:3
Fly food	Mouldable	NA	For 100 tubes: 1,125 ml H_2_O, 7.5 g agar, 55 g glucose, 50 g brewer’s yeast, 35 g flour, 25 ml nipagin (10% in ethanol), 4 ml propionic acid

## Results

### Development of *Megaselia scalaris* on different types of industrially obtained materials

We attempted to rear larvae on a wide variety of plastic, gum, wax and starch-based products (see ‘Materials and Methods’, Table 1). We monitored the duration of the different stages in all conditions (Table 2). Except for the paint treatments, adult flies had no problem surviving in all materials provided until egg hatching. In both paint treatments, adult flies died soon after the start of the treatment (but after egg laying), despite previous reports of *M. scalaris* feeding on liquid paint (McCrae, 1967). Based on intestine color, adult flies were feeding on latex based paint before death.

**Table 2:** Stage duration in different experimental conditions. Extent of stage duration (in days) of the longest surviving larvae, shortest successful larva to pupate, longest successful larva to pupate, shortest pupa to emerge and longest pupa to emerge in each of the tested conditions.

	Plastic bag	Starch bag	Adhesive tape	Nitrile gloves	Latex gloves	Latex based paint	Synthetic paint	Petroleum jelly	Wax ear plugs	Peripheral wax	Chewing gum	White tack	Modeling clay	Fly food
Max larva alive (not pupated)	13	26	17	5	13	18	NA	14	39	47	10	12	75	56
Min larva to pupa	NA	NA	NA	NA	NA	NA	NA	NA	29^a^	16	NA	NA	11	7
Max larva to pupa	NA	NA	NA	NA	NA	NA	NA	NA	29^a^	17	NA	NA	53	35
Min pupa to adult	NA	NA	NA	NA	NA	NA	NA	NA	NA	11^b^	NA	NA	10	13
Max pupa to adult	NA	NA	NA	NA	NA	NA	NA	NA	NA	11^b^	NA	NA	15	23

**Notes.**

NA indicates there is no value for that experiment.

a Refers to a single individual.

b Refers to a single individual.

### Effects on eggs

Flies laid eggs on all materials supplied, but the ability of the flies to complete their life cycle varied (see below). Only in the case of the synthetic paint did eggs fail to hatch.

### Effects on larvae

Larvae were obtained in all but synthetic paint treatments. Wax earplugs, periphery wax and modeling clay treatments produced larvae that reached the pupal stage. For the remaining materials (plastic carrier bag, potato starch carrier bag, adhesive tape, nitrile gloves, latex gloves, latex based paint, petroleum jelly, chewing gum, white tack), the larvae obtained died before pupation (Table 2).

The nature of the materials in terms of mouldability did not seem to have an effect on the chance of survival of the larvae, although wax and starch products did support longer living larvae than other study conditions (Table 2). In some cases we observed larvae feeding on dead eggs and larvae, suggesting that these individuals were surviving on carrion rather than the materials themselves. In other cases, larvae were obviously feeding on the food supplied as their guts were colored from the material (wax ear plugs, white tack, latex based paint and modeling clay).

We could also see air bubbles in the intestines or tegument of larvae in the periphery wax, wax earplugs, chewing gum, white tack, starch bag and adhesive tape experiments, although we do not know if this air was a consequence of the feeding itself, of the digestion of the food or an unrelated cause. Air bubbles have been reported before when *M. scalaris* have been submerged in a liquid environment, and they have been proposed to assist floatability (Harrison & Cooper, 2003). In our case, the larvae were not obligatorily submerged and we did not see bubbles in other conditions where water was also added to the media. Nevertheless we think these bubbles are equivalent to those observed in Harrison & Cooper (2003).

### Effects on pupae

We observed formation of pupae in the experiments fed on wax earplugs (1 pupa), periphery wax (2 pupae) and modeling clay treatments (21 pupae). Pupae from the experimental treatments were smaller than the ones obtained in the control (lengths ranging from 60% to 66% of the control pupae in modeling clay and periphery wax). In the case of the wax products the larvae that pupated may have been additionally feeding on the corpses of other larvae present in the tube. This may explain why only three pupae were obtained, although pupation did not occur for the other treatments where carrion feeding was observed.

### Effects on emerged adult flies

Adults were obtained from the periphery wax (1 emerged adult) and the modeling clay treatments (16 emerged adults). Modeling clay adults were smaller than the controls (see below). This was also observed in the case of the periphery wax adult (68% of the control size (1.87 vs. 2.27 mm)). Despite this, the single male fly from the periphery wax treatment was able to fertilize a control female and produce larvae. However these larvae only survived for two days under the same conditions. In the case of modeling clay, we could observe consistent formation of pupae and emergence of fertile adult flies, which we address in the next section.

### Effects on survival, life cycle and size of rearing *M. scalaris* on modeling clay

We found that eggs laid on modeling clay produced offspring that reached adulthood and were fertile. However, the amount of adult flies obtained per mating from the modeling clay condition was 84% less than that obtained from the controls (16 modeling clay, 98 controls). Since the amount of emerging larvae was similar between controls and modeling clay conditions, the difference is likely due to higher larval mortality in the modeling clay.

**Figure 1:**
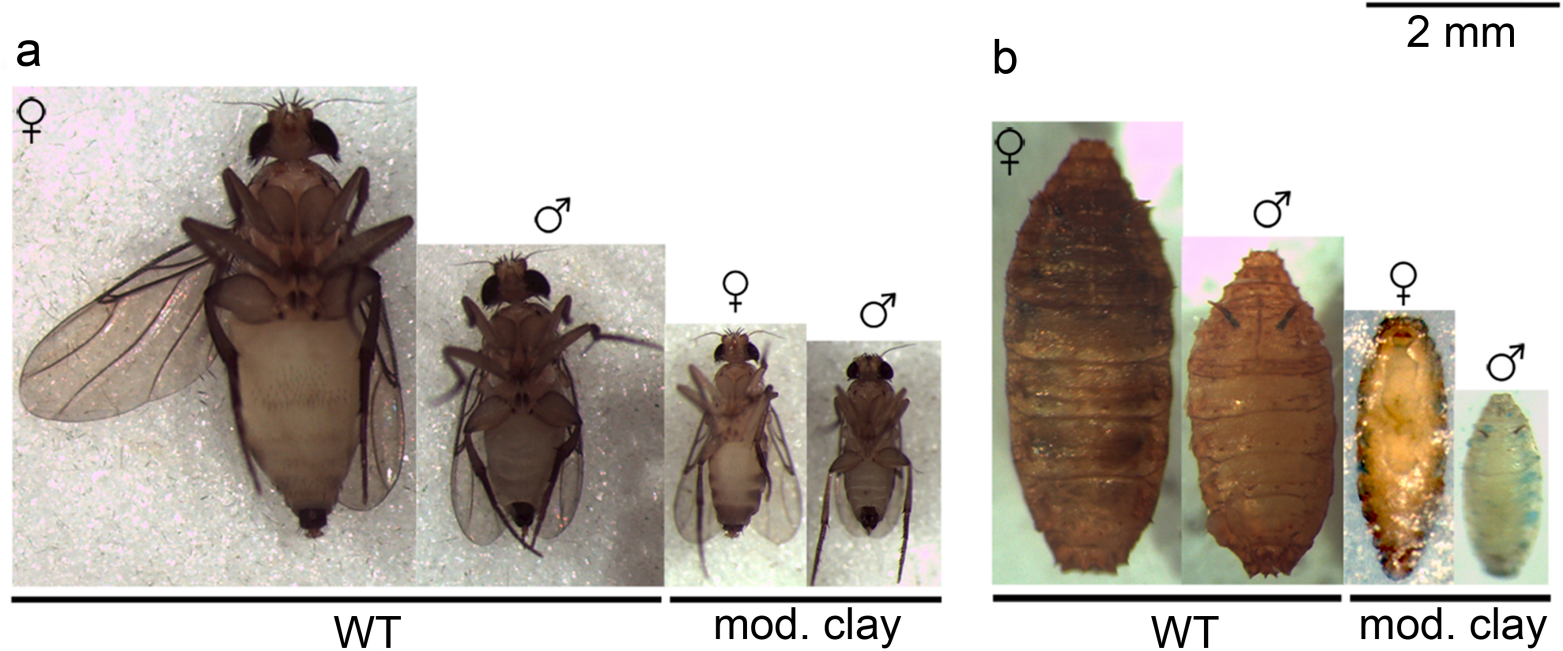
Size comparison between control and modeling clay animals. Size comparison between adult (A) and pupae (B) of control and modeling clay treatments. Male and female individuals for adults and pupae of control (WT) and modeling clay (mod. clay) treatments are shown. All adults showed in ventral view. All pupae showed in dorsal view except female in modeling clay treatment, in ventral view.

**Figure 2:**
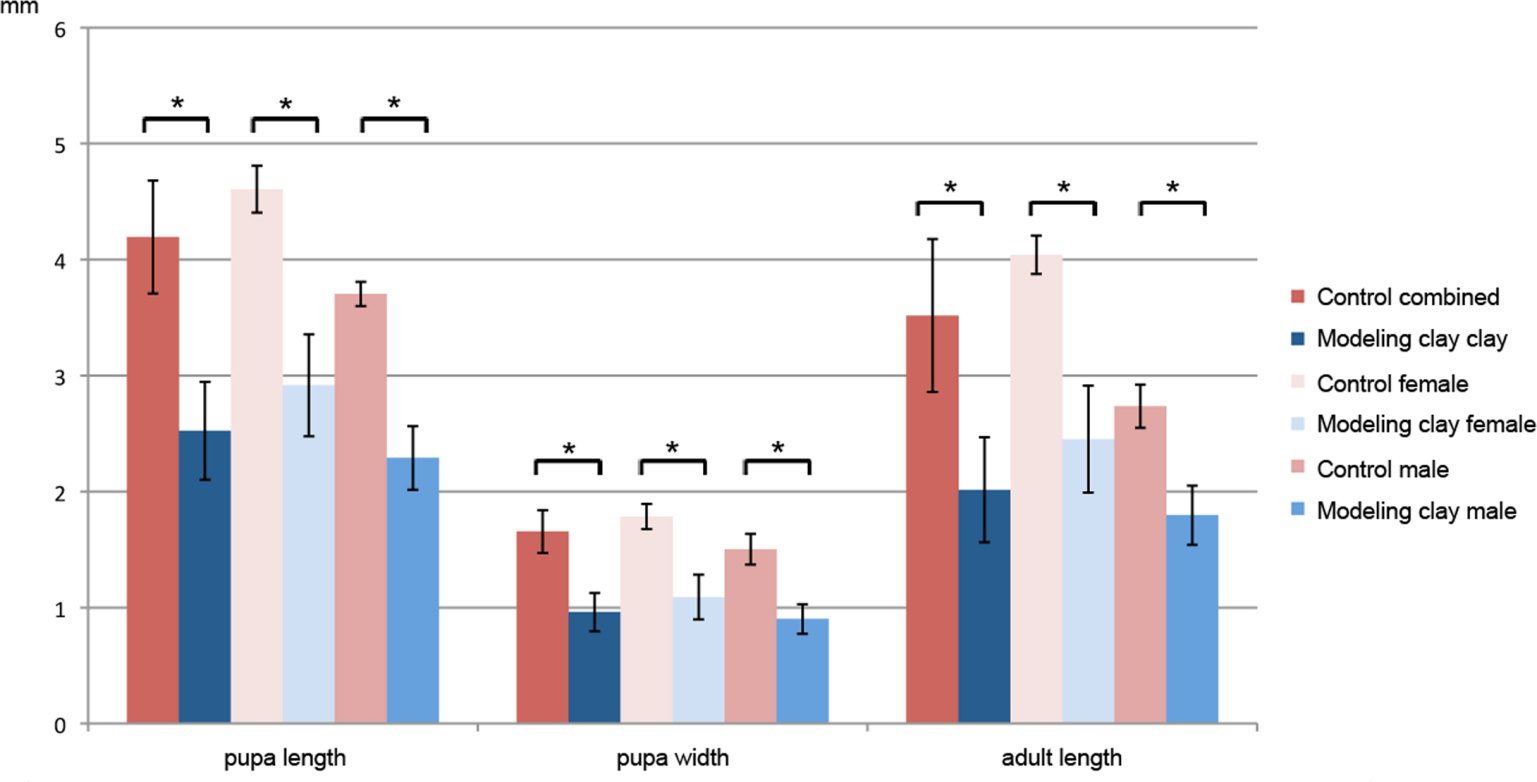
Adult and pupal size of *M. scalaris* reared on modeling clay and control diets. Mean pupal length and width and adult length (in mm) are shown for combined, female and male individuals in control and modeling clay conditions. Asterisks indicate significant differences between comparisons with all *p*-values <0.001.

We also observed that pupae and adults obtained from modeling clay-reared larvae are significantly smaller than those fed on standard food (female pupae are 63% of the control size, male pupae 61%, female adults 60% and male adults 65%; Figs. 1 and 2, see Table 3A for size averages). In all cases we find that females are significantly larger both as adults and pupae (control pupa males are 80% of female size, control adult males 67%; modeling clay pupa males 78% and modeling clay adult males 73%) (Fig. 1, 2 and Table 3A).

**Table 3:** Averages and standard deviations for size and duration measurements. (A) Size (in mm) of the pupa length, pupa width and adult length for control and modeling clay (Mod Clay) conditions. For each condition, the total (male + female), female and male Average ± Standard Deviation is given. (B) Stage duration (in days) of the larvae, pupa and adult stages for control and modeling clay (Mod Clay) conditions. For each condition, the total (male+female), female and male Average ± Standard Deviation is given.

**A**	**Size**	**Pupa length**	**Pupa width**	**Adult length**
	Control (total)	4.19 ± 0.49	1.65 ± 0.18	3.52 ± 0.66
	Control (female)	4.61 ± 0.20	1.78 ± 0.11	4.04 ± 0.17
	Control (male)	3.70 ± 0.10	1.50 ± 0.13	2.73 ± 0.19
	Mod clay (total)	2.53 ± 0.44	0.96 ± 0.17	2.02 ± 0.47
	Mod clay (female)	2.92 ± 0.44	1.09 ± 0.19	2.45 ± 0.46
	Mod clay (male)	2.27 ± 0.29	0.90 ± 0.14	1.79 ± 0.27
**B**	**Stage duration**	**Larvae**	**Pupa**	**Adult**
	Control (total)	14.8 ± 4.9	15.5 ± 2.0	26.7 ± 14.4
	Control (female)	15.8 ± 4.4	15.8 ± 2.4	32.6 ± 13.4
	Control (male)	13.3 ± 5.5	14.9 ± 1.1	17.6 ± 10.9
	Mod clay (total)	24 ± 12.2	12.4 ± 1.6	3.4 ± 2.1
	Mod clay (female)	17.2 ± 6.2	12 ± 1.9	5 ± 2.6
	Mod clay (male)	27.3 ± 14.8	12.2 ± 1.3	2.7 ± 2

**Figure 3:**
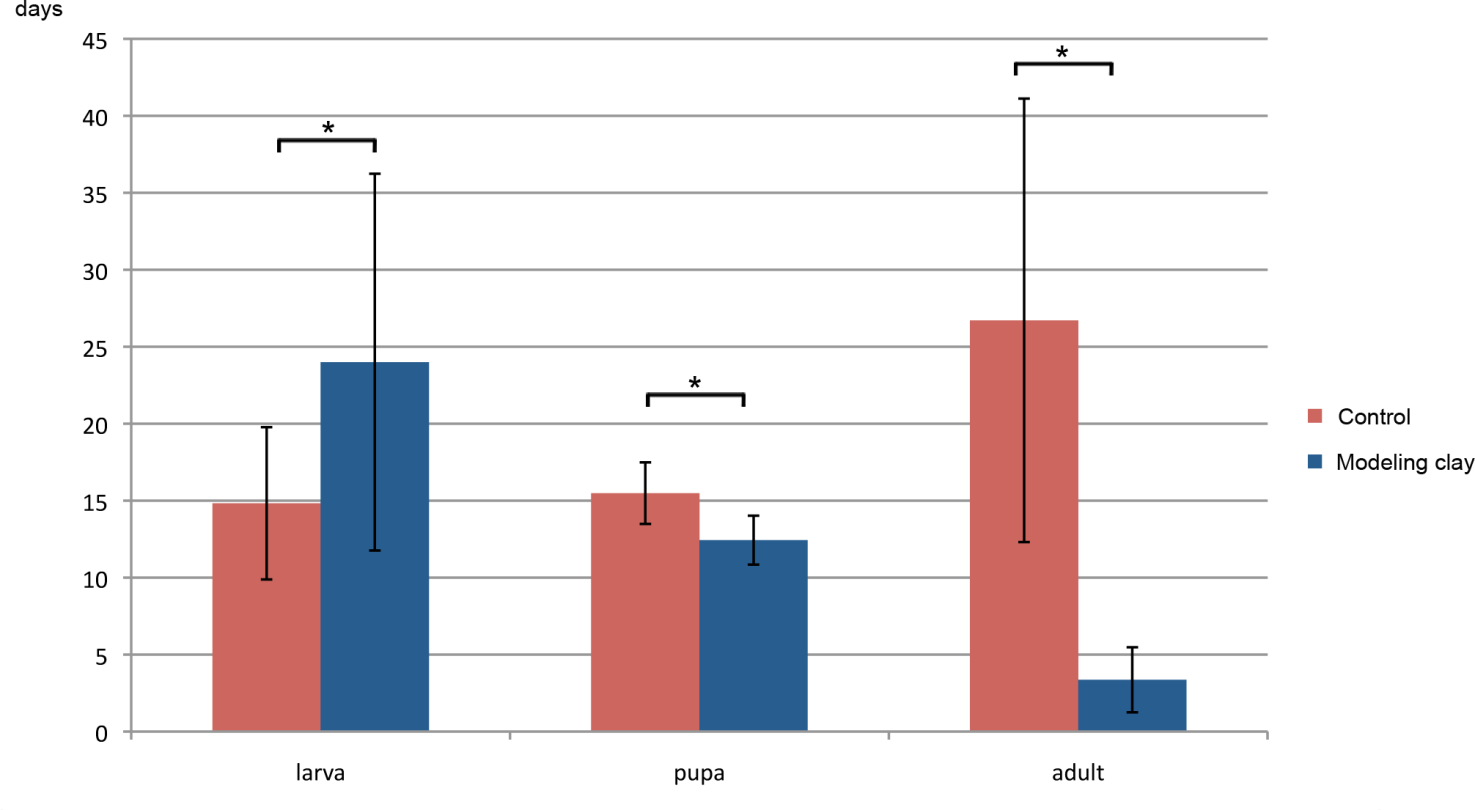
Stage duration of larvae, pupae and adult *M. scalaris* reared on modeling clay and control diets. Mean stage duration of larvae, pupae and adults (in days) are shown for individuals in modeling clay and control conditions. Asterisks indicate significant differences between comparisons with all *p*-values <0.01.

Developmental time in modeling clay fed animals differed significantly from that of controls at all stages (Fig. 3, see Table 3B for averages of stages duration). The time spent as larvae is extended in the modeling clay treatment (24 vs. 14.8 days), with higher variation between individuals than in the controls. The time spent as pupa for modeling clay fed animals is smaller to that of control individuals (12.4 vs. 15.5 days), and the variation is less pronounced than for the larvae, and similar to the controls. Adult lifespan is drastically reduced in modeling clay treated animals (3.4 days vs. 26.7 days). Only control adults have significant gender differences in the stage duration. Modeling clay treated flies may live too short to show any such trend.

## Discussion

The size of pupae and adult flies depends on the size of the larvae at the moment of pupation. Upon reaching the minimal viable size the larvae are able to survive metamorphosis (Nijhout, 1975). However, if they are starved before they attain this size, their larval stage is prolonged and they may die without going through to metamorphosis. To begin this process, the larvae must reach the critical weight, after which it will start producing the hormones needed for metamorphosis (Nijhout & Williams, 1974). In small larvae like *Drosophila melanogaster* (Meigen, 1830) these two weights are attained at the same time (Mirth, Truman & Riddiford, 2005). In the tobacco hornworm *Manduca sexta* (Linnaeus, 1763), when the critical weight is achieved, larvae continue feeding and growing for a few days. The size of the adult will therefore be determined by the critical weight and the growth after this has been attained. If, however, larvae were left to starve after reaching the critical weight, this would not delay metamorphosis (Davidowitz, D’Amico & Nijhout, 2003). This study also reported that larvae starved before reaching the critical weight take longer to pupate. In *D. melanogaster*, starvation after reaching the critical weight accelerates pupation, while larvae starved before eventually die without pupating (Mirth, Truman & Riddiford, 2005).

Diet restriction in *D. melanogaster* is usually attained by feeding standard food minus yeast (glucose and starch) (Tu & Tatar, 2003). In our modeling clay experiments, more starch is available in the food source than for the controls. However, it lacks free glucose or yeast, and is filled with inedible compounds that may affect the digestion of the starch (Table 1). In *D. melanogaster* experiments starvation generally starts at third instar, whereas in our experiments larvae were fed modeling clay from egg laying. In *D. melanogaster*, larvae died if starvation started before 72 h of growth (Beadle, Tatum & Clancy, 1938). Our experiments show that *M. scalaris* can survive in suboptimal food conditions from the beginning of the larval stage.

In *M. scalaris*, achieving the critical weight also presumably led to pupation. From the time when larvae reach the critical weight to the moment of pupation, the animals keep feeding and growing. In our modeling clay fed animals the rate of growth is smaller, with an increased time spent as larvae over the controls. Therefore growth achieved after critical weight is likely to be very small, and this may explain the small size of both pupae and adults compared to the controls. Critical weight and minimal viable size are probably, like in *D. melanogaster*, also attained at the same time in control animals. This also indicates that pupation is likely to happen with a very similar weight to the minimal viable size, hence these flies are likely to be close to the minimal size limit possible for this species. Indeed very few of the modeling clay fed larvae pupated, indicating that the minimal viable size was not achieved in the majority of larvae. which eventually died before pupation, as has been observed with starved *D. melanogaster* (Mirth, Truman & Riddiford, 2005). Interestingly, pupal success rate—pupae that eventually hatch to adults—in modeling clay treatments was higher than that of controls (76.2% vs. 53.8%). We think this could be due to the very restrictive selection for fitter larvae before pupation in modeling clay fed animals.

*Megaselia scalaris* diets influence the development of larvae and pupae both in terms of developmental time and size, as well as adult emergence and longevity (Idris, Abdullah & Lin, 2001; Harrison & Cooper, 2003; Zuha et al., 2012). The difference in pupal and adult size we observe is likely due to reduced nutrient content of modeling clay when compared to standard food. Larvae would stay at this stage until they reach the minimum viable size, which allows them to pupate successfully. Therefore the lower nutritional content of the food leads to an increased larval time to reach the minimum viable size or critical weight. We were not able to assess if the number of instar larvae was increased. In *Manduca sexta*, supernumerary instar larvae have been reported when middle instars molted before a minimal size was reached (Nijhout, 1975). Nevertheless, modeling clay fed larvae pupate before the normal size is reached, giving smaller pupae and adults. As discussed above, the larvae are unlikely to grow significantly after they reach the absolute minimum viable size, making this and the critical weight similar. This reduced size could also explain the faster pupation phase, since less tissue needs to be metamorphosed. *Megaselia scalaris* also displays sexual dimorphism in the size of the adults and pupae (Carareto & Mourao, 1988). In all cases we found that females are larger both as adults and pupae, indicating that sexual dimorphism is conserved in the smaller-sized modeling clay fed pupae and adults.

There is an almost universal extension of life span by reduced nutrient intake (Partridge, Piper & Mair, 2005). However, studies on the effects of limiting nutrition at the pre-adult (third instar larvae) stages, showed that it did not extend adult life span in *D. melanogaster* (Tu & Tatar, 2003; Zwaan, Bijlsma & Hoekstra, 1991). In *M. scalaris*, studies on different sources of food (meat and yeast supplements) have previously demonstrated that life expectancy of the adult positively correlates with better nutritional values (Idris, Abdullah & Lin, 2001). However, the results obtained in Idris, Abdullah & Lin (2001) differ from ours, as their adult flies were not permitted to feed, hence the life expectancy of our control flies was much higher (26.7 vs. 3.5 days). In our study, flies suffered a drastic reduction (more than 700%) of the adult life expectancy when fed on modeling clay. Since adult flies were feeding on the modeling clay (inferred by the coloured contents of their guts), we think that the reduced size of these flies, and not their capacity to eat, probably has a connection to their lower adult life expectancy. Despite reduced size and lower life expectancy, modeling clay fed flies were able to produce viable eggs, although these larvae did not pupate.

We cannot rule out that the inorganic toxic compounds found in modeling clay may have had a role in the reduced size and/or altered developmental time of the animals. Such compounds may also have functioned together with the poorer nutritional value and assimilation of the modeling clay.

## Conclusions

Modeling clay fed larvae have lower survival rates than controls and produce substantially smaller pupae and adults than those fed on standard food. Our results are in keeping with previous observations of insects reared under poor feeding conditions. In addition we find that sexual dimorphism is maintained in the nutrient-poor diet. The time spent as larvae is extended and more variable than in the controls, making the overall developmental time much longer. The length of the pupal stage is more similar to the controls, if shorter, and far less variable than the previous stage. Of those few larvae that pupate in the modeling clay treatment, more hatch successfully than in control treatment. We also find that life expectancy of adult flies is shorter for modeling clay fed larvae. In contrast to previous reports from 1967, we find that *M. scalaris* is unable to survive on paint, at least in its modern formulation, and additionally died when tested on most other industrial substrates.

## Supplemental Information

10.7717/peerj.1085/supp-1 File S1 Raw data for stages durations and size measurements for animals that completed their life cycle (STAGE DURATION) Stage duration of control, modeling clay and periphery wax animals. Averages for each group are given at the end of their list. Student’s T test values are given for a combination of parameters at the bottom of the list.
(SIZE) Sizes of control, modeling clay and periphery wax pupa and adults. Averages for each group are given at the end of their list. Student’s *T* test values are given for a combination of parameters at the bottom of the list.
